# Acute Hypersensitivity Pneumonitis With Hypoxic Respiratory Failure Secondary to Domestic Pigeon Exposure

**DOI:** 10.7759/cureus.117380

**Published:** 2026-10-03

**Authors:** Lahiru P Kariyawasam Katukoliha Gamage, Monisha, Abd Mohamed, Mai Abedalla, Robert Wekesa

**Affiliations:** 1 Acute Medicine, United Lincolnshire Teaching Hospitals NHS Trust, Lincoln, GBR; 2 Endocrinology and Diabetes, United Lincolnshire Teaching Hospitals NHS Trust, Lincoln, GBR

**Keywords:** avian antigen, bird fancier’s lung, ground-glass opacities, hypersensitivity pneumonitis, hypoxic respiratory failure, interstitial lung disease, pigeon exposure

## Abstract

Hypersensitivity pneumonitis (HP) is an immune-mediated interstitial lung disease caused by inhalation of environmental antigens in susceptible individuals. Bird fancier’s lung (BFL) is a well-recognised form of HP associated with exposure to avian proteins derived from feathers, serum, and droppings. Clinical presentation is variable and may range from relatively mild respiratory symptoms to severe hypoxaemia and respiratory failure. Early recognition is important because continued antigen exposure may result in persistent pulmonary inflammation and progression to chronic fibrotic lung disease. We report the case of a 27-year-old woman with no significant past medical history who presented with progressive dyspnoea over a one-month period, subsequently developing hypoxic respiratory failure requiring supplemental oxygen. She denied fever, chest pain, orthopnoea, productive cough, or peripheral oedema. She was a former smoker who had stopped smoking four years previously and had no relevant occupational exposures. Detailed environmental history identified prolonged indoor exposure to a rescued pigeon housed within the family kitchen. The patient required up to 10 L/min of supplemental oxygen. Computed tomography pulmonary angiography excluded pulmonary embolism but demonstrated diffuse bilateral ground-glass opacities with upper-lobe and perihilar predominance. Laboratory investigations demonstrated elevated lactate dehydrogenase and C-reactive protein, while renal function, liver function, complete blood count, and cardiac biomarkers were within normal limits. Extensive infectious and autoimmune investigations were negative. Serological testing demonstrated markedly elevated immunoglobulin G antibodies against pigeon serum, feathers, and droppings. The patient was treated with systemic corticosteroid therapy and strict avoidance of further avian antigen exposure. She subsequently demonstrated significant clinical improvement, with reduced oxygen requirements. The combination of a compatible exposure history, characteristic radiological findings, exclusion of important alternative diagnoses, positive avian-specific serology, and clinical response to treatment supported the diagnosis of acute hypersensitivity pneumonitis secondary to domestic pigeon exposure, consistent with bird fancier’s lung. This case highlights the importance of detailed environmental history taking in patients presenting with unexplained dyspnoea, diffuse pulmonary infiltrates, and hypoxic respiratory failure. Identification and avoidance of a causative domestic avian exposure may provide an important and potentially reversible therapeutic intervention.

## Introduction

Hypersensitivity pneumonitis (HP) is an immune-mediated inflammatory lung disease resulting from inhalation of environmental antigens in susceptible individuals. The immunopathogenesis of HP involves both immune complex-mediated (type III) and T cell-mediated delayed (type IV) hypersensitivity responses following inhalation of the inciting antigen. It encompasses a spectrum of clinical phenotypes, and the severity and pattern of disease may vary according to the nature and intensity of antigen exposure as well as individual susceptibility [1]. Bird fancier’s lung (BFL) is a well-established form of HP associated with exposure to avian proteins. Relevant antigens may be present in feathers, serum, droppings, and other avian-derived material [2,3]. Exposure may occur in occupational or domestic environments, and the diagnosis can be overlooked when environmental history taking is limited to employment-related exposures.

The underlying immunopathology involves both immune-complex-mediated and cell-mediated mechanisms, resulting in inflammation involving the small airways and pulmonary parenchyma [1,4]. Clinical manifestations are often nonspecific and may include dyspnoea, cough, fatigue, and other respiratory or systemic symptoms. Acute presentations can occasionally be severe and may result in significant hypoxaemia [3,4]. High-resolution computed tomography (HRCT) is an important component of the diagnostic assessment. Depending on the phenotype and stage of disease, imaging may demonstrate ground-glass opacities, poorly defined centrilobular nodules, mosaic attenuation, and air trapping [5,6]. Chronic exposure can result in fibrotic changes and progressive interstitial lung disease.

Identification of the causative antigen is central to diagnosis and management. Serum-specific immunoglobulin G (IgG) antibodies may provide evidence of sensitisation to a potential antigen, although positive serology alone is insufficient to establish HP and must be interpreted in conjunction with the clinical presentation, exposure history, and radiological findings [3,7]. We report a case of acute hypersensitivity pneumonitis associated with prolonged domestic pigeon exposure presenting with hypoxic respiratory failure. The case emphasises the diagnostic value of detailed environmental history taking in patients with unexplained respiratory deterioration and diffuse pulmonary abnormalities.

## Case presentation

A 27-year-old woman with no significant past medical history presented with progressive dyspnoea over approximately one month. Her respiratory symptoms gradually worsened and culminated in hypoxic respiratory failure requiring supplemental oxygen.

She denied fever, chest pain, orthopnoea, productive cough, and peripheral oedema. She was a former smoker who had stopped smoking four years previously. No relevant occupational exposures were identified. A detailed environmental history was subsequently obtained because the cause of her respiratory deterioration remained unexplained. This revealed prolonged indoor exposure to a rescued pigeon that had been housed within the family kitchen. The identification of this domestic avian exposure raised suspicion of bird-related hypersensitivity pneumonitis.

On presentation, the patient was tachypnoeic, with a heart rate of 113 beats/min, blood pressure of 143/72 mmHg, and respiratory rate of 25 breaths/min. Her oxygen saturation was 96% while receiving 7 L/min of supplemental oxygen; a room air oxygen saturation was not documented. Her oxygen requirement subsequently increased to 10 L/min. An arterial blood gas obtained while receiving 10 L/min of supplemental oxygen demonstrated a partial pressure of oxygen (PaO₂) of 10.2 kPa and oxyhaemoglobin saturation of 93.8%. A pre-oxygenation or room air arterial blood gas was not available. The cardiovascular and other systemic examinations were unremarkable. Computed tomography pulmonary angiography was performed to investigate the cause of her hypoxic presentation. Pulmonary embolism was excluded. The scan demonstrated diffuse bilateral ground-glass opacities with upper-lobe and perihilar predominance (Figures 1-2).

**Figure 1:**
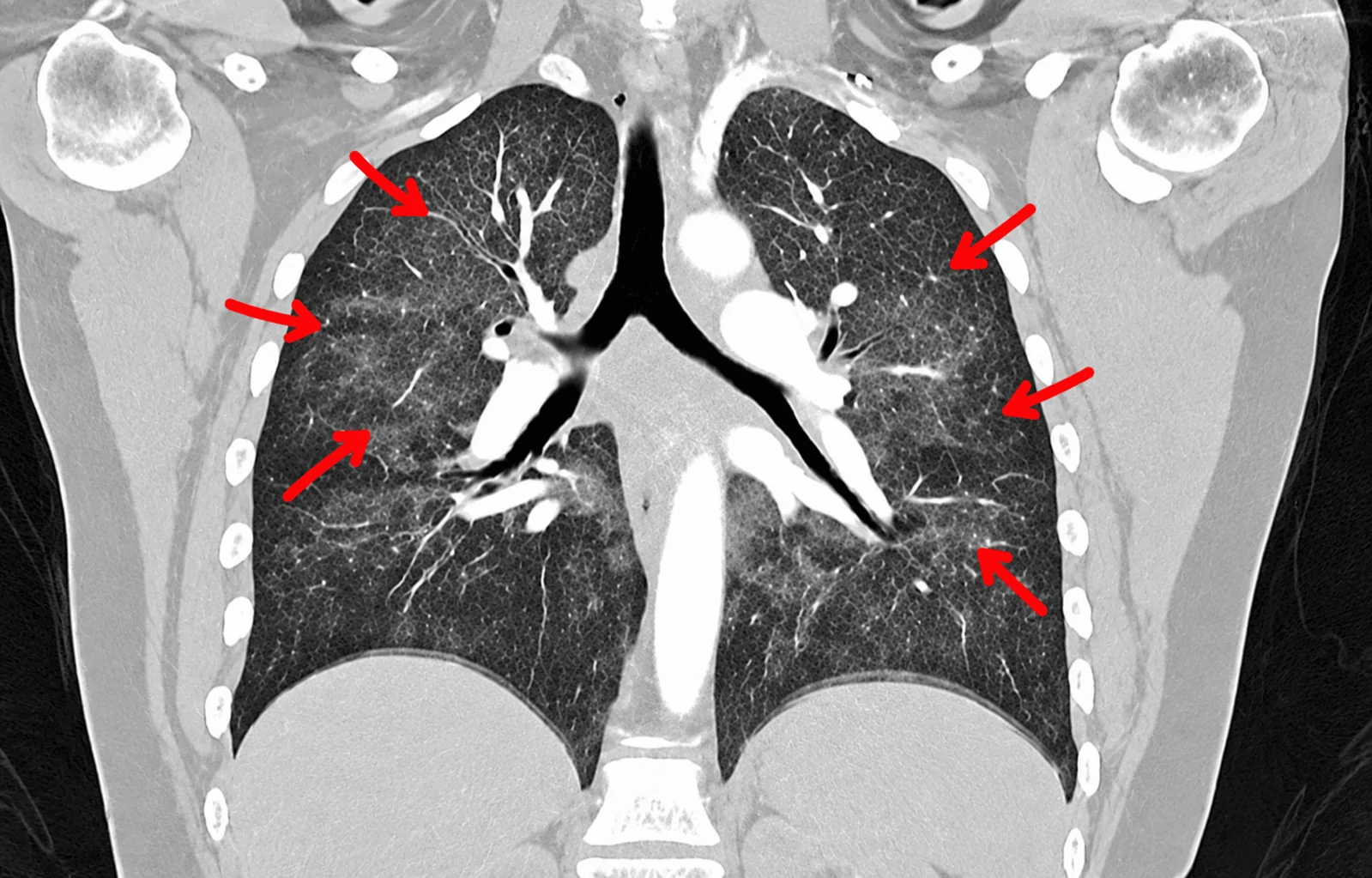
Coronal computed tomography pulmonary angiography image Red arrows indicate representative bilateral areas of ground-glass pulmonary attenuation.

**Figure 2:**
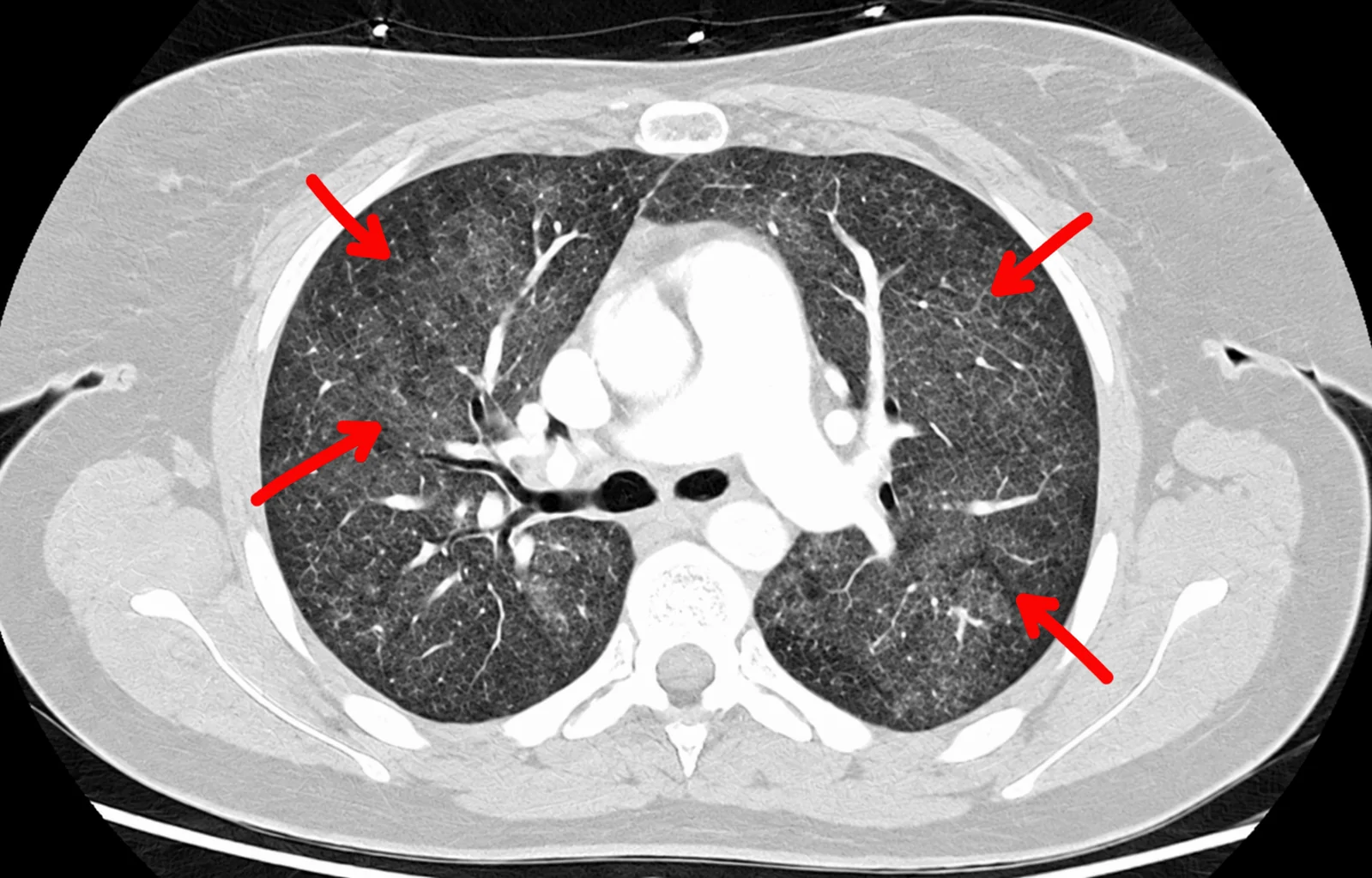
Axial computed tomography pulmonary angiography image Red arrows indicate representative bilateral areas of ground-glass pulmonary attenuation.

Laboratory investigations demonstrated an elevated lactate dehydrogenase level of 423 U/L and a C-reactive protein level of 43 mg/L. Renal function, liver function, complete blood count, and cardiac biomarkers were within normal limits. Autoimmune screening, including antineutrophil cytoplasmic antibody testing, was negative. A marginally elevated anticardiolipin IgG level of 22 U (reference range 0-20 U) was identified. In the absence of supportive clinical features and with otherwise negative autoimmune investigations, this isolated low-level elevation was not considered explanatory for the pulmonary presentation. Repeat anticardiolipin antibody testing was not performed during follow-up. Respiratory viral and atypical infection screening was also negative (Tables 1-2).

**Table 1:** Routine laboratory investigations during the acute illness eGFR, estimated glomerular filtration rate; INR, international normalised ratio

Investigation	Day 1	Day 4	Reference range
Creatinine (µmol/L)	75	70	45-84
Urea (mmol/L)	4.8	6.6	2.5-7.8
eGFR (mL/min/1.73 m²)	>90	>90	90-200
Bilirubin (µmol/L)	8	4	0-21
Alanine aminotransferase (U/L)	11	10	0-33
Alkaline phosphatase (U/L)	68	60	30-130
Lactate dehydrogenase (U/L)	423	—	135-214
C-reactive protein (mg/L)	43	12	0-5
Hemoglobin (g/L)	143	134	117-149
Platelets (×10⁹/L)	263	306	150-400
White cell count (×10⁹/L)	8.6	6.8	4.3-11.2
INR	1.1	—	0.9-1.1

**Table 2:** Infectious, autoimmune, and hypersensitivity investigations ENA, extractable nuclear antigen; IgG, immunoglobulin G; IgM, immunoglobulin M; PCR, polymerase chain reaction; RSV, respiratory syncytial virus

Investigation	Result	Reference range/expected result
Coronavirus PCR	Not detected	Not detected
Influenza A PCR	Not detected	Not detected
Influenza B PCR	Not detected	Not detected
RSV PCR	Not detected	Not detected
Mycoplasma pneumoniae IgM	Not detected	Not detected
β2-glycoprotein 1 antibody, IgG	<6.4	0-20 U
β2-glycoprotein 1 antibody, IgM	3.5	0-20 U
Antinuclear antibody	Negative	(-)
Myeloperoxidase antibody	<3.2	0-16 U
Proteinase-3 antibody	<2.3	0-20 U
Cardiolipin antibody, IgG	22.0	0-20 U
ENA antibody screen	Detects antibodies to Ro, La, Sm, Jo-1 and Scl-70	(-)
Glomerular basement membrane antibody	<2.9	0-20 U
C3	1.57	0.90-1.80 g/L
C4	0.34	0.10-0.40 g/L
IgG to budgerigar serum/feather/dander	>200	0-40 mg/L
IgG to pigeon serum/feather/dander	>200	0-40 mg/L
IgG to parrot serum/feather/dander	185	0-40 mg/L

Serological testing demonstrated markedly elevated IgG antibodies against pigeon serum, feathers, and droppings (Table 2). In the context of prolonged indoor pigeon exposure, compatible radiological findings, hypoxic respiratory failure, and negative investigations for important alternative diagnoses, the findings supported a diagnosis of acute hypersensitivity pneumonitis secondary to domestic pigeon exposure.

The patient was commenced on oral prednisolone 40 mg once daily and was advised to strictly avoid further exposure to pigeon-related antigens. Prednisolone was continued at 40 mg once daily for two weeks, followed by 30 mg once daily for two weeks and then 20 mg once daily. Following treatment and antigen avoidance, she demonstrated significant clinical improvement with reduced oxygen requirements. Supplemental oxygen was successfully discontinued within three days of treatment, and the patient was discharged on day five without a requirement for supplemental oxygen. At respiratory review after three months on prednisolone 20 mg once daily, the dose was reduced to 5 mg once daily for a further month and then discontinued. Following completion of corticosteroid therapy and continued antigen avoidance, repeat CT imaging demonstrated complete radiological resolution without evidence of fibrotic change. The final diagnosis was hypersensitivity pneumonitis secondary to domestic pigeon exposure, consistent with bird fancier’s lung.

## Discussion

Bird fancier’s lung is a recognised form of hypersensitivity pneumonitis resulting from inhalation of avian antigens. It can occur following exposure to birds in both occupational and domestic environments. The relevant antigenic material may include proteins derived from feathers, serum, and droppings [2,3]. Morell et al. described a series of 86 patients with bird fancier’s lung and demonstrated the clinical heterogeneity of the disease [2]. The presentation may vary according to the pattern and intensity of exposure, and acute, subacute, and chronic forms have been described. The diagnosis therefore requires consideration of the clinical syndrome in combination with the exposure history and appropriate investigations. 

In the present case, the patient developed progressive dyspnoea over one month and subsequently developed significant hypoxaemia requiring up to 10 L/min of supplemental oxygen. The severity of the presentation illustrates that avian-related HP should not be regarded solely as a chronic or mild respiratory condition. Acute HP can produce substantial hypoxic respiratory impairment and may initially resemble other causes of diffuse pulmonary disease [3,4]. A particularly important feature of this case was the identification of the causative exposure within the patient's home. The rescued pigeon was housed inside the family kitchen, resulting in prolonged indoor exposure. This illustrates the importance of asking specifically about domestic environmental exposures rather than restricting the history to occupational factors. 

The radiological findings were characterised by diffuse bilateral ground-glass opacities with an upper-lobe and perihilar predominance. Ground-glass attenuation is a recognised feature of HP, although it is not specific to the condition. Other HRCT features that may support HP include poorly defined centrilobular nodules, mosaic attenuation, and air trapping [5,6]. The radiological findings must therefore be interpreted within the clinical context. In this patient, the presence of a significant avian exposure and compatible imaging findings increased the likelihood of HP, while investigations for several important alternative diagnoses were negative. Pulmonary embolism was excluded by CT pulmonary angiography. Infectious investigations, including respiratory viral and atypical infection screening, were negative. Autoimmune screening, including ANCA, was negative, and renal function, liver function, complete blood count, and cardiac biomarkers were within normal limits. These findings helped reduce the likelihood of several alternative causes of hypoxic respiratory failure and diffuse pulmonary infiltrates. Serological testing demonstrated markedly elevated IgG antibodies against pigeon serum, feathers, and droppings. Specific IgG testing can provide useful evidence of exposure to a potential inciting antigen. However, antigen-specific IgG should not be interpreted as diagnostic in isolation because sensitisation does not necessarily indicate clinically significant HP [3,7]. 

The diagnosis of HP requires integration of the exposure history, clinical findings, imaging, and other relevant investigations. Current international guidance emphasises a structured and multidisciplinary approach, with particular attention to identifying potential causative exposures and interpreting serum-specific IgG results within the broader clinical context [6]. In this case, the markedly elevated avian-specific antibodies were particularly supportive because they corresponded with a clear and significant environmental exposure and a compatible clinical and radiological presentation. The negative infectious and autoimmune investigations further strengthened the overall diagnostic assessment. Antigen avoidance is a central component of HP management. Continued exposure to the causative antigen can result in ongoing pulmonary inflammation and may contribute to progression towards chronic fibrotic disease [6,8]. Therefore, identifying the relevant antigen is not only diagnostically important but also directly influences treatment. 

The patient was advised to avoid further exposure to pigeon-related antigens and was treated with systemic corticosteroids. Following treatment and antigen avoidance, she demonstrated significant clinical improvement, including a reduction in oxygen requirement. The clinical response was consistent with the inflammatory nature of the disease and provided additional support for the overall diagnosis. Systemic corticosteroids are frequently used in clinically significant or acute inflammatory presentations of HP, although treatment decisions should be individualised according to disease phenotype and severity [8,9]. The principal therapeutic objective remains removal of the inciting antigen. An important concern in HP is progression from inflammatory disease to chronic fibrotic lung disease if exposure persists. Chronic fibrotic HP may be associated with impaired lung function and poorer outcomes [1,6,8]. Early recognition and removal of the causative exposure are therefore important. 

This case demonstrates how a relatively simple but detailed environmental history can be decisive. The patient's domestic pigeon exposure might not have been recognised as clinically significant without specifically asking about her home environment. Once the exposure was identified, it provided a coherent explanation for the clinical presentation, radiological abnormalities, and positive avian serology. The case also demonstrates the importance of avoiding over-reliance on any single diagnostic finding. Ground-glass opacities are nonspecific, and positive avian-specific IgG demonstrates sensitisation or exposure rather than proving HP. The diagnosis in this case was supported by the combination of prolonged pigeon exposure, compatible clinical presentation, radiological findings, exclusion of alternative diagnoses, positive avian-specific serology, and improvement following corticosteroid therapy and antigen avoidance. Contemporary ATS/JRS/ALAT guidelines classify HP as nonfibrotic or fibrotic rather than using the historical acute, subacute, and chronic categories [6]. The final diagnosis was nonfibrotic hypersensitivity pneumonitis secondary to domestic pigeon exposure, presenting with acute hypoxic respiratory failure and consistent with bird fancier’s lung.

## Conclusions

Hypersensitivity pneumonitis should be considered in patients presenting with otherwise unexplained hypoxaemic respiratory illness, particularly when imaging demonstrates diffuse ground-glass abnormalities. A detailed environmental history is essential for identifying potentially overlooked antigen exposures, and early antigen avoidance combined with appropriate treatment can result in substantial clinical improvement. This case demonstrates nonfibrotic hypersensitivity pneumonitis secondary to domestic pigeon exposure presenting with acute hypoxic respiratory failure, with complete radiological resolution and no evidence of fibrotic change following corticosteroid therapy and sustained antigen avoidance.
